# Strategic Planning of Rural Development Based on Foresight Methodologies

**DOI:** 10.1155/2020/5195104

**Published:** 2020-02-20

**Authors:** Rasul Gusmanov, Almir Askarov, Milyausha Lukyanova, Vitaliy Kovshov, Eugene Stovba

**Affiliations:** ^1^Department of Economics and Management, Federal State Budgetary Educational Establishment of Higher Education “Bashkir State Agrarian University”, Ufa 450076, Russia; ^2^Department of Informatics and Economics, Birsk Branch of Federal State Budgetary Educational Establishment of Higher Education “Bashkir State University”, Birsk 452450, Russia

## Abstract

The purpose of the study is to establish scientific rational for the use of the foresight methodology in the strategic planning of rural development. The scientific novelty of the study is determined by the development of an algorithm for strategic planning of rural development based on the foresight methodology and by the formation of a set of practical recommendations for the use of foresight tools at the municipal level of management in rural areas. The paper states that modern foresight methodology is quite flexible and multifaceted. It can be widely applied at different hierarchical levels of management. In our research, we consistently analyzed foresight projects and programs used in the rural management and development forecasting. The use of a systematic approach in combination with foresight technologies allows developing strategic plans for the rural areas development from the perspective of improvement of their economic and social component. The research presents the foresight algorithm of the rural development strategic planning and its implementation mechanism at the municipal level. The main components of the foresight testing procedure of the rural areas economic development were determined on the example of such a classic agricultural region of the Russian Federation as the Republic of Bashkortostan. The results of a comprehensive foresight analysis of alternative scenarios of the rural development have been formed. We summarized that the foresight technologies should be used as a system tool for the formation and implementation of the strategy of the sustainable rural areas development. The main results of the study include summarizing the experience of foresight studies on the rural areas development; design of an algorithm of strategic planning of the rural areas development based on the foresight methodology; the formation of alternative scenarios of the rural areas development at the regional level.

## 1. Introduction

Today, there is an increased interest of the scientific community to the problems of the use of foresight technologies in the strategic planning of the development of space territorial systems. Classical planning methods are mainly focused on “guessing,” while the result of foresight researches is a map of the future, which visualizes the socioeconomic space of rural areas and allows considering alternative ways to achieve the desired result [1]. As Patrick Becker emphasizes, foresight is a process of active knowledge of the future, a vision of mid-term and long-term prospects in science, economy and society. This process is aimed at supporting and mobilizing joint efforts to make and implement relevant decisions [2]. At the same time, according to Pierce, the process of future planning consists of three main stages: creation of the idea for future, future concept formulation, and the definition of the laws of the development of the “vision of the future” concept when comparing with the dynamics of the “present” [3].

In such countries as the United States, Canada, countries of the European Community, and Japan, the concept of “smart specialization” is widely practiced. The concept allows focusing not on individual sectors of the agricultural economy, but on certain activities of economic entities (agroformations). General methodological principle of foresight and its development in foreign countries is the involvement of different social forces such as scientific community, business structures, civil society, and representatives of state municipal authorities in the process of promising strategies discussing and making forecasts for the rural areas development [3]. The main development imperative of the modern foresight concept determines a more active use of the knowledge of the expert community involved in the development of foresight projects [4].

In economically developed countries various methods are used to develop foresight projects. They are quantitative and qualitative methods. Economic and mathematical modeling, analysis and forecasting of indicators, and extrapolation of trends belong to quantitative methods. Qualitative methods include role-playing games, morphological analysis, interviews, literature reviews, and planning of alternative scenarios. To design a rural development strategy, any researcher can certainly use different combinations of foresight methods and technologies. At the same time, each country or region uses its own combination of foresight methods (tools) to design a development strategy. Methodological approaches are improving constantly. Procedures and techniques are processed and corrected. All this ensures the validity of rural development prospects prediction [5]. In this case, one method is chosen to be dominant, and the others complement the overall picture of the rural areas future development. Thus, as noted by Miles, researchers widely use the “success scenario” method, which is based on the formation of the desired image of the future when implementing the “only successful” scenario [6].

In the USA, foresight is developed within individual agricultural sectors. There, foresight coprograms (participatory programs) are effectively applied. American foresight can be called institutional management of the future. It is implemented primarily as a management technology. In the United States, when developing a rural development strategy, they widely use “triple helix” models, which mean the method of “reverse forecasting or back-casting.” At the same time, the “triple helix” model is considered as a conceptual platform for the formation of expert community consisting of representatives of government, business, and science. In this case, the following principles of foresight research are implemented: involvement (commitment) and communication of social forces, focusing on a long term and coordination [7].

These models reflect the stages of the strategic planning of particular mechanisms to achieve the desired future. Experts with “foresight” thinking and having extraordinary personal qualities are actively attracted in Canada when implementing successful foresight projects. A key component of Canadian foresight seminars is the identification of those major problems that, according to experts, will determine the development of rural areas over the long term in the future [8].

In the European Union (EU), according to the adopted Lisbon strategy, all the countries entering the EU must form their regional foresights. The Modern European version of foresight is based primarily on the inertial development of rural areas and, as foreign researchers note, there is no ontology in its methodological basis [5, 9, 10]. At the same time, most of the European foresight methods are extremely formalized and meaningful. They basically do not contain a prognostic element [11–13]. In Germany, foresight tools are actively used to assess and predict rural development [14, 15]. Perspective German foresight projects are represented by a system of tracking (monitoring system). The system helps to search and spread new relevant information by a mechanism of strategic dialogues between experts [16].

The basis of Brazilian rural development foresight is determined by the research agenda of the National Council for Scientific and Technological development and by such foresight programs as “Brazilian Technology Foresight Programme,” “Prospectar,” and “Brasil 3 Moments Project” [17].

In Japan, long-term foresight projects for rural development (with an outlook for mainly 30 years) are very popular. They are coordinated by the Council for Scientific and Technological Policy (CSTP). It should be noted that Japanese foresight focuses primarily on two key areas: long-term trends review and determining of promising technologies and monitoring of the current state of rural development [18]. In China, two major foresight initiatives have been successfully implemented in recent years. They are as follows: “Scientific and Technological Foresight 2020” and “Innovations 2030: Road Map for Development.” Scenario methods, critical technologies, and road mapping are widely used to study them [19].

It should be emphasized that today contextual or “open” foresight is developing in foreign countries which takes into account environmental factors. Moreover, special attention is paid to the discussion of opportunities and stimulating fundamental changes in the existing paradigm of rural development. At present, in the context of the development of planning and program documents for sustainable development of rural areas, systemic and synergistic foresights are successfully combined. There is the integration of foresight and “competitive intelligence” [20, 21].

In the Russian Federation, foresight is a relatively new phenomenon. The new paradigm of the rural development strategic planning in the Russian Federation determines the development of a methodology that would be based on the use of foresight. At the same time, in scientific literature there is practically no scientific research results devoted to the rural development strategic planning based on the use of foresight methodology. In recent years, researches made by Russian scientists are mainly devoted to the development of foresight projects that solve the problems of forming strategies for the development of industries and large cities. In Russian practice of foresight research, there is no single organizational mechanism for interaction between municipal authorities and the local expert community. Etalon tools for the foresight research on the rural municipalities development have not been worked out.

A characteristic feature of Russian foresight is its great attention paid to ontological issues of the development of the studied objects. The practice of Russian foresight has become widespread since the middle of the first decade of the 21st century. At the regional level, foresight projects were formed in the Irkutsk, Saratov, Perm, and Krasnoyarsk regions. A preforesight project was organized in the Republic of Sakha. In the Republic of Bashkortostan the formation of a foresight project in 2005 was primarily associated with the choice of innovative priorities and the development of a strategy for innovative development of the region [22–24]. The main work methods were critical technologies in combination with SWOT analysis, as well as focus groups formation and expert survey [25, 26].

Today, there is a growing interest of the Russian scientific community to the problems of foresight application. However, many methodological issues of foresight of rural development remain unresolved, open to discussion, and insufficiently studied. In our opinion, among these problems are foresight integration and road mapping when developing strategic plans for sustainable development of rural municipalities, formation of strategies based on these plans, and assessing the effectiveness of foresight projects, as well as the lack of detailed methodological approaches to the development of foresight studies at the rural level. Today, more accurate forecasts are required. They should be based on the real functional possibilities of rural entities with greater influence of management structures on their future development. At the same time, unlike traditional forecasting, the result of foresight research is not only the definition of prospects and strategic guidelines, but also the development of practical measures for their implementation. As noted by the editor-in-chief of the Russian “Foresight” scientific journal Gokhberg, in comparison with other forecasting methods foresight is not only a system of expert methods of alternative assessment, but also a certain combination of forecasting process and results [27]. The solution of these topical issues is not a trivial task and necessitates the use of a systematic approach to this study.

The purpose of the study is to establish scientific rational for the use of the foresight methodology in the strategic planning of the rural areas development. The research objects are to design an algorithm of strategic planning of the rural areas development based on the foresight methodology as well as the formation of alternative scenarios of the rural areas development at the regional level.

## 2. Materials and Methods

The issues of rural areas development in the region under current conditions are determined by a versatility and dynamism of different scientific approaches and methods to be applied for solving these issues [28–30]. These circumstances determine a special role of the foresight methodology and a systematic approach for designing rural development strategy. The use of these modern scientific methods allows assessing rural development from the perspective of prospective improvement of the economic and social component of the rural areas development. The algorithm of strategic planning of rural development designed with the application of foresight methodology is based on the formulation of the rural areas mission, hierarchical goal-setting, analysis of socioeconomic problems of rural development, and their ranking.

When making a foresight research, we take into account the natural resource potential, local specific conditions that determine the development of agricultural production, and location of social facilities and infrastructure in rural areas. Foresight research is based on a comprehensive approach, which involves examination of all elements of the studied territorial system (rural areas in total), including strategic objectives and the definition of relationships between the elements of the system.

We have to subscribe to the opinion of Heinz, who thinks that one of the most effective methods of foresight is scenario planning. This method helps to make such a choice of options for change in the studied organization, which in the future will allow achieving the goals [31]. When designing promising courses of rural areas development, it is necessary to use a scenario approach instead of strictly deterministic “solid” forecasts. As the academician of the Russian Academy of Sciences Altukhov emphasizes, “the scenario approach is one of the most effective system tools for strategy development, as its application provides a better understanding of the situation and assessment of potential threats and identification of favorable opportunities for determining the most likely activities of all spheres of the agroindustrial complex, as well as increasing the level of their adaptation to changes in the external environment” [32].

Integrated use of foresight technologies as well as scenario approach application allows identifying the most favorable in socioeconomic terms rural areas as “potential growth points” of the agricultural sector. Thus, special attention can be paid to depressed rural areas which are a kind of “incubators of poverty” at the zonal level. Scenario approach specifies choice diversity and alternativeness of a trajectory of the sustainable development strategy [33, 34]. Alternative scenarios for the development of rural areas are formed on the basis of foresight technologies with the involvement of the expert community.

Expert's study of strategic directions of the rural areas development involves two rounds. Pre-poll training of experts is carried out before each round. In order to get valid results before the second round, the results of the first round were analyzed and registered. Then, experts were acquainted with them. After the second round, the final analysis was carried out, and the results of the entire expert survey were presented.

Round 1 consists of the following stages.

  Stage 1: pre-poll training, during which the following issues were identified: the definition of specific tasks and conditions of expert polls, revealing sources of information which can be used for a deeper study of problems, searching for specialists and experts which would take part in foresight sessions and conferences in order to make the most objective evaluation, and a competent long-term forecast of the rural areas development of the region. At the same time, development directions and topical issues are formulated. These issues become “statements” for the subsequent expert poll. Criteria for issues hierarchy are developed.
  Stage 2: development of a polling sheet (questionnaire), containing indicators and strategic directions of rural development, which are subsequently subject to expert evaluation.
  Stage 3: a survey of experts. Experts were divided into several focus groups. Seminars were given for each particular group at which the study issues were discussed and analyzed independently of other expert groups. “Brainstorming” and the method of expert panels used within each working group helped identify the main trends in the long-term development of rural areas.
  Stage 4: expert analysis of a preferred strategy of the sustainable development of rural areas. The purpose of this analysis was to determine the most realistic and attractive strategy for the sustainable development of rural areas in future.
  Stage 5: processing and analysis of the survey results. Statistical analysis of the study results was made to establish expert's feedback.

Round 2 included a secondary expert survey to clarify the summarized opinion of an expert group and to improve the experts' opinion consistency referred to the group assessment. The results of the previous survey round helped to get more information which experts can use during repeated rounds. This allows excluding or minimizing the interest's influence peddling of individual experts. Expert assessments differed from each other. We studied these differences and revealed previously unnoticed aspects of the problem which allowed fixing the attention of the expert community on probable consequences of the development of the analyzed socioeconomic situation in the considered rural areas.

After having made expert surveys and analyzed the results of the quantitative study, group discussions, “round tables” were held, the purpose of which was to discuss the results of the quantitative study, determine the current situation, and identify the problems, conditions, and expected results of this study. Subsequently, the results of all focus groups were summarized and presented for discussion. Final decisions were made at the final foresight conference.

In this case, the foresight methodology is based on the targeted identification and the use of knowledge of experts. Among them are representatives of the executive and legislative regional authorities, scientific and research community members, public representatives, media, businessmen, and heads of rural municipalities of the Republic of Bashkortostan. As part of the study, we conducted a survey among experts (scientists and public and agribusiness representatives) on the prospects for the development of rural areas of the region. There were three focus groups of experts in total. Each group consisted of 15–20 people.

## 3. Results

**Figure 1:**
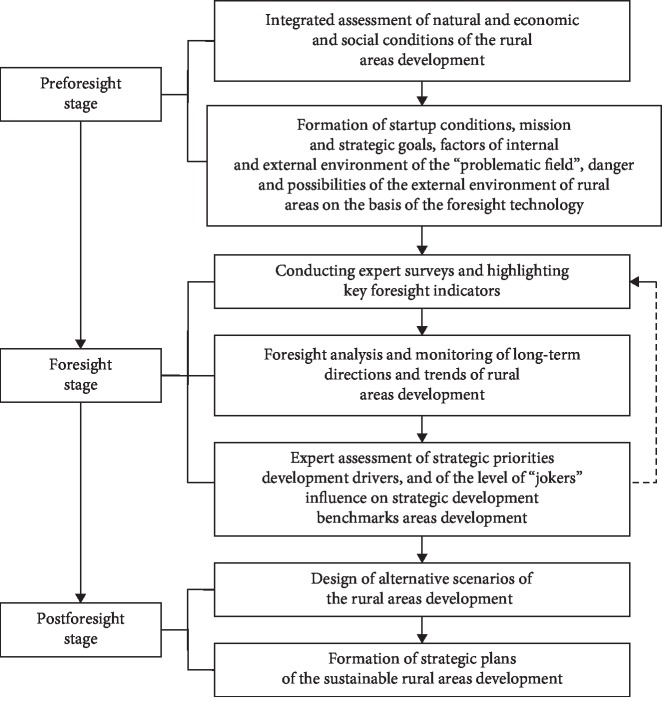
Algorithm of strategic planning of the rural areas development based on the foresight methodology.

The nature of the municipal foresight is determined, on one hand, by the need to respect the interests of key actors of regional development. On the other hand, it is conditioned by necessary interconnection of strategic priorities for the rural areas development of in the future. The algorithm of the rural development strategic planning designed on the basis of the foresight methodology is presented in Figure 1.

Development and initialization of the foresight research includes the following:

(I) “Preforesight stage”:
  (1) Formation of the concept of the strategic rural areas development of the region:
    (i) Formulation of strategic goals, subgoals and tasks, key foresight indicators, its typology, form and methodology, priority areas and time horizon; analysis of foreign and domestic experience in the use of foresight; development of a foresight project management plan (methods to be used are bibliometric method, sources review, and scanning)
    (ii) Identification of key problems of rural development to be solved as part of a foresight project: analysis of these problems, their relevance and socioeconomic significance, possible limitations (method to be used is a system analysis)
    (iii) Analysis of the current development of rural areas in the region, highlighting the main trends, directions and potential of their development, the definition of “problem” rural areas, scientific justification of the use of a system of methods to eliminate imbalances in the socioeconomic development of rural areas, development of a comprehensive forecast of rural development (methods to be used are trend extrapolations, environmental scanning, SWOT analysis)
(II) “Foresight stage”:
  (2) Development and initialization of the foresight research:
    (i) Analytical stage: formation of the object, foresight project essential conditions (targets); identification of the widest possible range of experts and stakeholders
    (ii) Organizational stage: selection and detailing of expert groups according to the foresight trends and the level of their professional competence and awareness (method to be used is focus groups formation)
    (iii) Information support: collection and processing of statistical, analytical, forecast, scientific, technical, and regulatory information in order to provide experts with it in the required volume and within the established time (methods to be used are analysis of open information sources and analysis of information flows)
    (iv) Identification of foresight stakeholders and rural development goals setting taking into account the expert survey of stakeholders: analytical subdivision of goals them into subgoals and analysis of the general goal and its components (methods to be used are mapping of stakeholders and “objective tree” construction)
    (v) Formation of a foresight “common field,” identification of problem and unknown zones (uncertainty areas) in the future, and “growth points” and management decision-making: at this stage trends in the development of rural areas which can be predicted are identified. Based on the coordination of stakeholders' expert opinions, possible future scenarios are created (methods used: preparation of expert and public panels, expert surveys, and cluster analysis)
    (vi) Analysis of expert knowledge, expert analysis of the most promising areas of social and economic development of rural areas with regard to the established time horizon using foresight methodology, research among the population, discussion of multistep expertise, coordination of strategic plans for rural development with representatives of the executive power, production and business, and development and structuring of a set of practical foresight research measures (methods used: Delphi expert survey and converse scenario planning)
    (vii) Structuring of the obtained results, scenario formation and development strategies based on the identified during the foresight analysis “signals of the future”; that is, those socioeconomic priority areas which, according to experts, will be the most important sources for future development of rural areas; designing a “window of opportunity,” i.e., time intervals in the future, during which critical management decisions should be taken (methods: “brainstorming” and modeling)
(III) “Postforesight stage”:
  (3) Organization of public discussion of the foresight project layout, foresight conference, monitoring and adjustment of the strategy of socioeconomic development of rural areas, and evaluation of the proposed measures effectiveness: the application of this method helps to determine the lag degree of rural municipalities, to develop measures aimed at improving competitiveness of agricultural economy and solving the most important social problems of the population of rural areas of the region (methods used: foresight conferences and benchmarking)
  (4) Scenario development is based on the analysis of opportunities and construction of alternative trajectories of the rural areas development in the region (methods used: extrapolation of trends, construction of scenarios, and road mapping). Alternative scenarios for the development of rural areas are formed on the basis of foresight technologies with the involvement of expert community. As a result, the methodology of foresight is formed, in which the system process is implemented: “goal-tasks-state-alternative scenarios-execution.”

The foresight study allowed forming alternative scenarios for the development of rural areas. They are as follows: pessimistic (conservative) scenario, safe (inertial) development scenario, and sustainable development scenario. Development scenarios are formed with regard to the natural and climatic conditions and the achieved level of production of the area. They are focused on the practical implementation depending on the prevailing environmental conditions and factors of the internal state of the rural areas. Let us consider the main parameters of these scenarios in more detail.

1. The pessimistic scenario is based on the concept of a conservative forecast, the parameters of which reflect severe restrictions and the shift of the agricultural economy in rural areas to more unfavorable conditions compared to the actual state. This scenario determines extensive development of rural areas and its design is carried out taking into account the preservation of certain destructive trends and crisis phenomena.
2. When considering the pessimistic scenario, it is assumed that negative factors affecting the development of agricultural production will increase. A significant reduction in state support (subsidies, governmental grants) and financing of agricultural sectors leads to a consistent deterioration in the financial condition of agricultural organizations. In the pessimistic scenario, unfavorable ratio (disparity) of prices for industrial and agricultural products will remain.
3. Thus, slow growth of the population income implies low demand for agricultural products sold by local rural producers. The pessimistic scenario assumes the use of forecast indicators of agricultural organizations of rural areas when making calculations.
4. The scenario of safe development is designed taking into account a certain determinability of rural areas economic development. At the same time, the formation of the scenario parameters is determined by the achievement of certain development stability of rural areas and is based on the continuation of existing trends in their economic development.
5. This scenario provides for the inertial strengthening of the existing positive trends, including those that lead to the end of farm production decline in agricultural organizations. The improvement of certain elements of the rural economy is of gradual and evolutionary character.
6. The scenario of safe development is focused on slow economic recovery of agricultural sector and is designed to maintain moderate state support for agricultural organizations. Income of the rural population will slightly increase, which will maintain moderate growth in the demand for agricultural products. When considering the scenario of safe development, the average annual actual indicators of dynamic development of agricultural organizations in rural areas are taken as a basis.
7. The sustainable development scenario is based on the potential of a significant increase in the efficiency of agricultural production in the future. Parameters of the scenario are focused on the intensive development of agricultural production in most agricultural organizations. This scenario is designed to form potential “points of growth” within rural areas, that is, agricultural organizations that have significantly improved their economic state using internal reserves and by virtue of the production structure optimization of crop and livestock industries.

Consideration of the sustainable development scenario includes economic regulation and implementation of extensive state support for agricultural producers directly at the municipal level of management. This shall cause the creation of a favorable market environment for the development of crop and livestock production within the considered rural areas. Stimulating and increasing the investment prospects of agricultural sectors will highlight priority areas of the agroindustrial complex development of rural areas and, in particular, crop and livestock industries.

Under the sustainable development scenario, the income of the rural population is expected to grow quite rapidly. Accordingly, the growth of real income and improvement of the quality and standard of living of the population will contribute to increased demand for agricultural products. The sustainable development scenario takes into account the possibility of creation of positive factors that determine the increase in agricultural production. For this kind of scenario, they mainly use forecast or sometimes actual indicators of the development of agricultural organizations in rural areas in the models.

On the basis of the formed alternative scenarios, production value of food and its consumption for rural areas of the non-Chernozem zone of the region have been designed. Scenario variants are based on the results of foresight analysis and model solutions, as well as on the forecasting of possible volumes of the main food products made by agricultural formations (Table 1).

In accordance with science-based nutrition standards, the population of rural areas will be fully provided with bread, potatoes, vegetables, meat, and milk in the short-term. Forecast calculations show that, in the short term, the production of eggs and vegetable oil will be insufficient in comparison with the norms of the World Health organization and will not be able to fully satisfy consumers demand for this products.

In the medium term, optimal parameters of the designed development scenarios ensure full provision of the population with the necessary production value of bread, potatoes, vegetables, meat, and milk. In accordance with the cost of living and with the norms of the World Health Organization, by 2022 the level of self-sufficiency of the population with eggs will be more than 100%, and with vegetable oil less than 17%, respectively.

Production value and consumption of agricultural products in rural areas according to the formed scenario of sustainable development for the period up to 2030 are presented in Table 2.

In the long term, the implementation of the parameters of the sustainable development scenario determines the full provision of the population of rural areas with all basic food products except vegetable oil.

Thus, the definition of perspective directions of agricultural market development allows forming positive prerequisites aimed at sustainable development of rural areas. Foresight monitoring of municipalities allows forming a forecast assessment of the actual situation in rural areas where food supply of residents is on a low level. The use of the scenario approach and foresight technologies increases the validity of the developed forecasts, as well as allowing developing measures to regulate the trajectory of sustainable development of rural areas.

## 4. Discussion

In contrast to previous foresight projects presented after having studied publications of other scientists [20, 21], in our research we did not consider alternative scenarios of the territorial entities development based on model solutions. The main method of previous studies when considering a particular region was the use of critical technologies. In this article, based on foresight, we have justified the use of the scenario approach. The main parameters of the three alternative scenarios of rural development are variants of pessimistic, safe, and sustainable development.

According to the published sources [22, 23], the researchers during their foresight studies did not take into account the key factors and parameters reflecting the work of agricultural sector at the regional level. Special attention in the previous foresight studies [20, 21] was primarily paid to the innovative development of the region. In our research we made the scenarios of the agrifood sector development of rural areas. These scenarios show possible ways for rational provision of the population with food in accordance with scientifically based nutrition standards.

We have developed a series of methodological recommendations. From one side, they can improve the quality of the planning and forecasting activities of municipal authorities. From the other side, they will expand the horizon period of the strategic planning when determining the strategic parameters of the sustainable rural areas development. The use of alternative scenarios and foresight methodology is scientifically justified and allows determining the priority factors affecting the production of agricultural products.

Multifunctional development of rural areas will be determined on the basis of increasing agricultural production volumes, improvement of the economic efficiency of production activities of agricultural organizations, creation of new jobs in rural areas, and, finally, significant increase in the living standards of the rural population.

Practical evaluation of the algorithm of strategic planning proposed in the article allows concluding on its rational use for the design of rural development strategy. Methodological approaches and provisions for strategic planning of rural development which have been designed using the foresight methodology can be used for creating road maps and strategic plans for socioeconomic development of rural municipalities.

## 5. Conclusions

Thus, the author's concept of strategic development of rural areas of the region is based on the following:

- Formulation of strategic goals, subgoals and tasks, key foresight indicators, its typology, form and methodology, priority areas and time horizon; analysis of foreign and domestic experience in the use of foresight; development of a foresight project management plan;
- Identification of rural development key problems to be solved as part of a foresight project: analysis of these problems, their relevance and socioeconomic significance, and possible limitations;
- Analysis of the current development of rural areas in the region, highlighting the main trends, directions, and potential of their development, the definition of “problem” rural areas, scientific justification of the use of a system of methods to eliminate imbalances in the socioeconomic development of rural areas, and a comprehensive forecast of rural development.

The results of the study are as follows:

1. Theoretical approaches and positions on the strategic planning of the rural areas development on the basis of the foresight methodology have been generalized. The international experience has been systematized. The opportunities of the strategic planning of rural areas with the use of foresight technologies have been assessed.
2. There has been designed an algorithm of the strategic planning of rural development on the basis of the foresight methodology, consisting of preforesight, foresight, and postforesight stages.
3. When using foresight technologies, alternative scenarios for the development of rural areas have been formed.
4. On the basis of the scenario approach, the projects of production value of food and its consumption for the considered rural areas have been designed.

Strategic planning of rural development on the basis of foresight methodology will improve the quality of a decision-making process at the municipal and regional levels of government. It will become possible to develop in time measures aimed at reducing imbalance between different layers of rural residents, to reduce rural society stratification and to make odds even in the rural areas society. The use of modern foresight technologies together with the system analysis not only helps to quickly diagnose the current state of rural areas but also contributes to spot “bottlenecks” and to reveal problems in the system of strategic management of material resources, to model the adoption of optimal management decisions. The results of the study may be of practical importance in the development and adjustment of plans and programs for the rural areas strategic development.

## Figures and Tables

**Table 1:** Consumption and production of basic agrofood products in the non-Chernozem zone of the region over time, thousand tons.

Food products	Consumption		Production^*∗*^		
	Cost of living	Norms of the World Health Organization	Scenarios of development		
			1	2	3
Short-term prospects (until 2022)					
Bread	44	53	399	408	413
Potatoes	39	42	290	332	342
Vegetables	47	61	63	76	79
Meat	23	31	64	67	70
Milk	132	157	510	521	542
Eggs, million pcs.	89	106	99	100	104
Seed oil	3.8	5.7	0.6	0.7	0.8

Mid-term prospects (until 2025)					
Bread	44	52	367	419	434
Potatoes	40	42	212	341	362
Vegetables	47	61	52	78	87
Meat	23	30	62	682	76
Milk	131	155	502	527	593
Eggs, million pcs.	88	105	95	103	112
Seed oil	3.8	5.7	0.5	0.8	1.0

Long-term prospects (until 2022)					
Bread	43	51	346	428	452
Potatoes	38	41	152	347	391
Vegetables	46	60	43	81	94
Meat	22	30	59	70	87
Milk	129	153	497	531	642
Eggs, million pcs.	86	103	92	106	126
Seed oil	3.7	5.6	0.4	0.9	1.2

^*∗*^The volume of production of farms of all categories is taken into account in forecast calculations. ^*∗∗*^Development scenarios: 1: pessimistic, 2: safe development, 3: sustainable development.

**Table 2:** Production and consumption of agrifood products in rural areas of the non-Chernozem zone of the region under the scenario of sustainable development for the long-term prospects, thousand tons.

Indicators	Food products						
	Bread	Potatoes	Vegetables	Meat	Milk	Eggs, million pcs.	Seed oil
Consumption in accordance with science-based nutrition standards							
Cost of living	43	38	46	22	129	86	3.7
The norms of the World Health Organization	51	41	60	30	153	103	5.6

Sustainable development scenario							
Production^*∗*^	452	391	94	87	642	126	1.2

^*∗*^The volumes of production of farms of all categories are taken into account in forecast calculations.

## Data Availability

The data supporting the findings of this study are available from the corresponding author upon request.
